# Goutweed (*Aegopodium podagraria* L.)—An Edible Weed with Health-Promoting Properties

**DOI:** 10.3390/molecules30071603

**Published:** 2025-04-03

**Authors:** Kamila Dębia, Małgorzata Dzięcioł, Agnieszka Wróblewska, Katarzyna Janda-Milczarek

**Affiliations:** 1 Department of Biology, Parasitology and Pharmaceutical Botany, Faculty of Pharmacy, Medical Biotechnology and Laboratory Medicine, Pomeranian Medical University in Szczecin, 72 Powstańców Wielkopolskich Street, 70-111 Szczecin, Poland; kamila.debia@gmail.com; 2 Department of Chemical Organic Technology and Polymer Materials, Faculty of Chemical Technology and Engineering, West Pomeranian University of Technology in Szczecin, 42 Piastów Avenue, 71-065 Szczecin, Poland; malgorzata.dzieciol@zut.edu.pl; 3 Department of Catalytic and Sorbent Materials Engineering, Faculty of Chemical Technology and Engineering, West Pomeranian University of Technology in Szczecin, 42 Piastów Avenue, 71-065 Szczecin, Poland; agnieszka.wroblewska@zut.edu.pl

**Keywords:** goutweed, ground elder, *Aegopodium podagraria* L., biologically active compounds, bioactivity

## Abstract

Goutweed *(Aegopodium podagraria* L.) is a species of medicinal perennial in the celery family (Apiaceae), also considered an edible plant with medicinal effects and high nutritional value. In traditional folk medicine, it was known as a remedy for gout (arthritis) and also used to relieve rheumatism or sciatica. The botanical characteristics, occurrence, nutritional composition, and traditional and present-day applications of this plant are discussed. Furthermore, the important specific plant metabolites including organic acids and their derivatives, flavonoids, coumarins, polyacetylenes and terpene components of essential oil are presented and their biological activity is described. The valuable medicinal properties of *Aegopodium podagria* L. include anti-inflammatory, antirheumatic, antioxidant, antibacterial, antifungal, diuretic, sedative and protective effects on the kidneys and liver. The aim of this paper was to describe, on the basis of the available literature, the chemical composition, bioactivity and health-promoting properties of this wild edible plant. The information obtained is described and summarized in tables.

## 1. Botanical Characteristics

Goutweed (*Aegopodium podagraria* L.), also known as ground elder, is a medicinal perennial in the celery family (Apiaceae) [1,2,3], which includes more than 3500 species distributed worldwide [4,5,6]. Its numerous representatives are edible plants, among which we can distinguish vegetables (carrots, parsley, parsnips, celery) and spices (dill and fennel, anise, coriander, chervil, cumin) [4,5]. Goutweed is the only representative of the genus *Aegopodium* found in Poland [7]. The Latin name of this species is derived from the Greek words *aix, aigos* (goat, goat) and *podion* (little foot); it refers to the shape and arrangement of the leaf segments of the groundsel, which resemble the shape of a goat’s hoof [8,9]. Both the names “goutweed” and “*podagraria*” reflect the plant’s application, as in the Middle Ages it was used as a remedy for arthritis [10,11]. Goutweed is an expansive ground cover with a long growing season, emerging very early in the spring. The height of the plant (60–90 cm) depends on the site, soil richness and irrigation [12]. The parts used are leaves, fruits/seeds and rhizomes as well as flowers [13]. The best time to harvest the leaves is from April to May. Flowering occurs between June and July; the flowers can be used as an edible decoration and for making infusions. Fruiting occurs in July–August [14,15,16]. The ripe fruits are harvested by cutting off the aboveground parts of the plants [13]. Underground rhizomes act as storage organs for reserves for the next growing season and allow asexual reproduction and regeneration from small fragments left in the soil [7,17,18,19,20]. At the beginning of the growing season, the rhizomes use mainly carbohydrates in the form of starch, fructosans and disaccharides, the content of which decreases during the vegetation period and increases in autumn before the plant goes into dormancy [17,21,22]. The starch content of rhizobacteria changes with the development of leaves and rhizomes, so its content is closely linked to the growth stages of this perennial plant [17]. Rhizomes can be harvested in the fall by digging them up and drying [13]. Goutweed is an invasive plant species found mainly in the temperate zone of the northern hemisphere [5,21]. It is native to Europe, Siberia, the Caucasus, Kazakhstan and Central Asia, but now it is also growing in North America, Australia and Japan [7,14,19,23,24,25]. Its primary habitats are shady and moist forests, thickets, ditches and gardens [7,24].

## 2. Nutritional Composition

Goutweed is characterized by a diverse and rich chemical composition, including both primary and secondary metabolites, which are synthesized by plants for special functions, e.g., protection against some adverse external factors (UV radiation, pests). It is a source of carbohydrates such as glucose and fructose, lectins and glycoprotein-binding carbohydrates. These substances are a valuable source of nutrients and can contribute to beneficial health effects for the human body [26,27]. Consumption of goutweed can provide a valuable dietary supplement of antioxidant compounds that can also be found in more common sources, such as fruits and vegetables [28]. The above-ground parts provide vitamin C, lutein, neoxanthin, violaxanthin, antheraxanthin and zeaxanthin carotene as well as coumarins, polyacetylenes and flavonoids (quercetin and kaempferol) [29]. It is also a source of vitamins, macronutrients and micronutrients [1,30,31]. Goutweed contains amino acids (arginine, histidine, leucine, lysine, threonine, valine and methionine) and various elements (calcium, potassium, iron, zinc, magnesium, aluminum, molybdenum, vanadium, copper, chromium, manganese, cobalt, titanium, gallium, phosphorus, silicon, boron and fluorine) [26,27]. Infusions of this plant have been shown to be a source of dietary fluoride. The fluoride content of the infusion depended on the morphological part used (rhizome, leaves, flowers and seeds) and the temperature of the water (25 °C, 70 °C, 80 °C and 90 °C) used to prepare the infusion [32]. Research in Poland has shown that ground elder, like other medicinal plants, can also be a source of radioactive elements such as ^210^Po and ^210^Pb. Nevertheless, according to the authors, its consumption, taking into account both the content of these elements and the amount of herbs consumed, should not have a negative effect on health [33]. Unsaturated and saturated fatty acids and other organic acids have also been identified in the herb [1,30]. The nutritional composition of the aerial parts of goutweed is shown in Table 1.

## 3. Biologically Active Compounds and Their Properties

Goutweed is a rich source of various biologically active substances. Its biological activity is mainly attributed to the presence of compounds belonging to the group of flavonoids, phenolic acids (including various derivatives of hydroxycinnamic acids), tocopherols, tannins, coumarins, polyacetylene compounds, components of essential oils, and micro- and macroelements, among others [36,37,38,39].

### 3.1. Organic Acids

**Figure 1:**
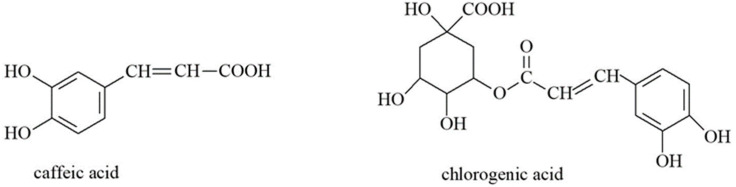
The chemical structures of caffeic acid and chlorogenic acid.

Organic acids are natural or synthetic organic compounds with specific physiological activities. The most common in plants are oxalic, formic, citric, fumaric, malic, succinic, acetic and ascorbic acids [40]. These compounds are primarily responsible for flavor, in addition to their various beneficial effects on human health. Among others, they exhibit antioxidant, anti-inflammatory and antibacterial effects and can regulate the intestinal microbiome and metabolism [41]. Phenolic acids such as caffeic, chlorogenic, gallic, protocatechuic and ferulic acids are known for their antioxidant activity [42]. The content of organic acids in plants is related to respiration, transpiration and various biochemical processes, and can vary depending on the phenophase of the plant [43]. Goutweed extracts have been shown to contain derivatives of hydroxycinnamic acids, e.g., caffeic acid and chlorogenic acid (Figure 1) [39,44]. Small amounts of palmitic, pelargonic, myristoleic, caprylic and capric acids have also been found in this plant [45], as well as sinapic, vanillic, α-resorcylic and protocatechuic acids [46].

### 3.2. Flavonoids and Coumarins

**Figure 2:**
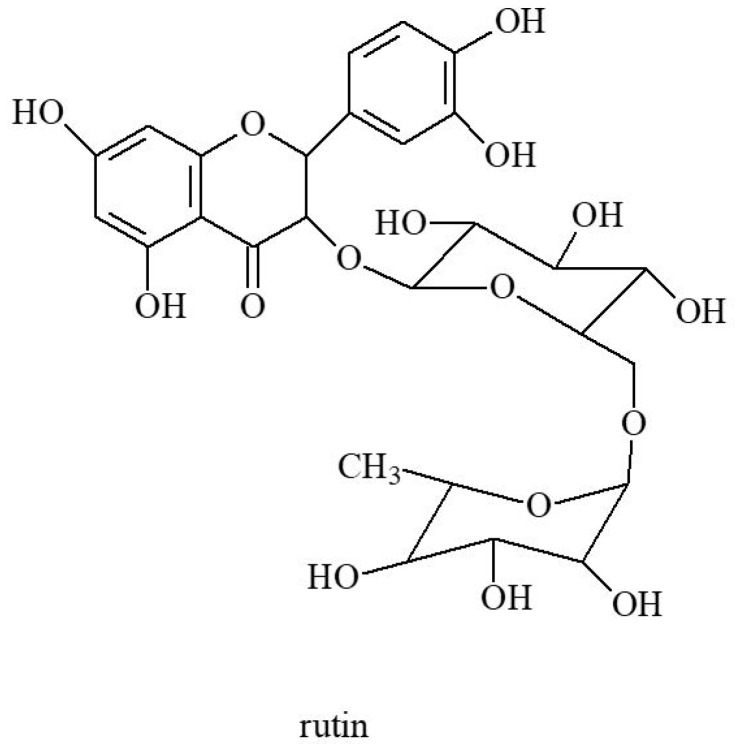
The chemical structure of rutin.

**Figure 3:**
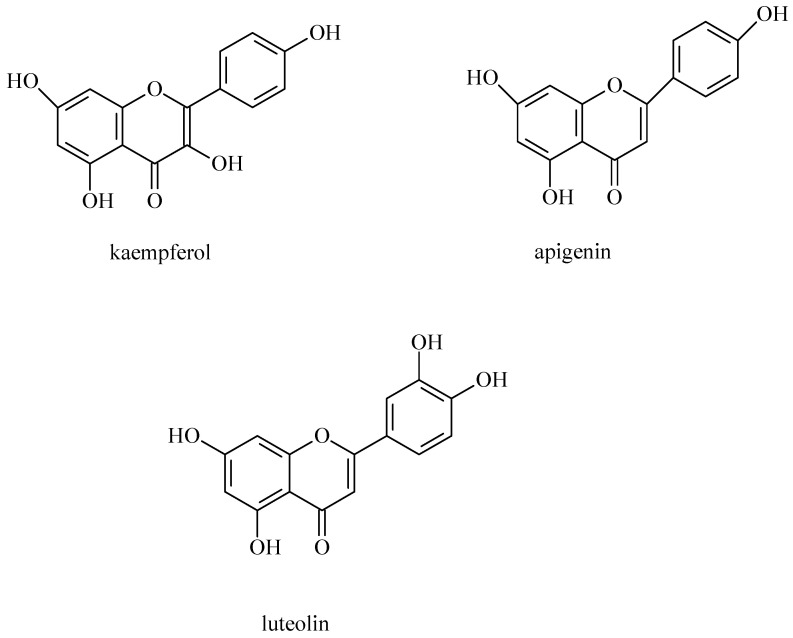
The chemical structures of kaempferol, apigenin and luteolin.

**Figure 4:**
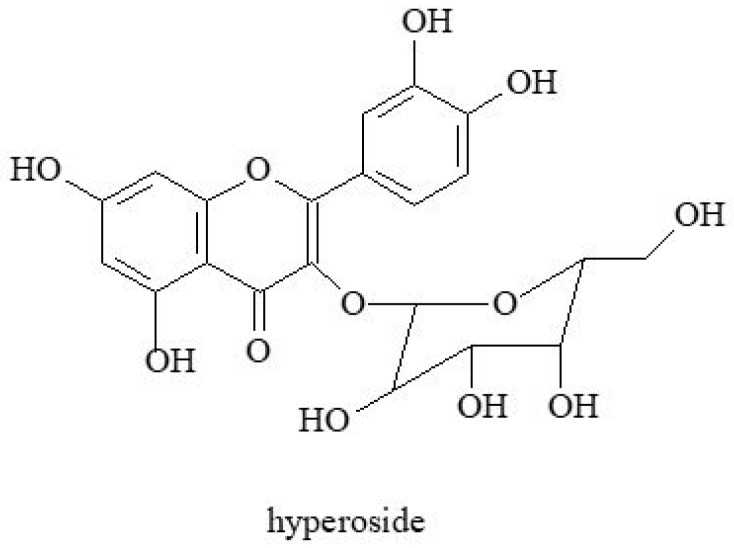
The chemical structure of hyperoside.

Flavonoids and coumarins are an important group of biologically active compounds [1]. Flavonoids belong to a subclass of natural polyphenols of a relatively low molecular weight and include rutin (Figure 2), kaempferol, apigenin and luteolin found in goutweed (Figure 3) [47]. Among furanocoumarins, angelicin and apterin have been shown to be present in goutweed [48]. According to Augspole [46], ground elder leaves are a source of kaempferol, luteolin, quercetin, rutin and catechin hydrate. Extracts from common goutweed also contain flavonoid glycosides and quercetin derivatives: 3-*O*-galactoside of quercetin (hyperoside)—Figure 4, 3-*O*-glucoside of quercetin (isoquercetin), kaempferol 3-galactoside (trifolin) and an unspecified rhamnoglucoside [26]. The antioxidant activity of compounds from this group may help reduce the risk of chronic diseases (e.g., cardiovascular disease, neurodegenerative disease, autoimmune diseases, obesity, diabetes, cancer) and other health conditions that are mediated by reactive oxygen species [28,49].

### 3.3. Polyacetylenes

**Figure 5:**
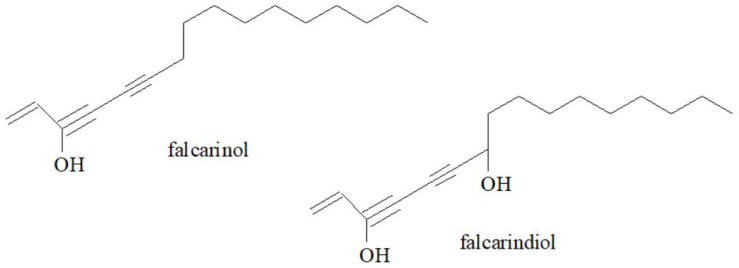
The chemical structures of falcarinol and falcarindiol.

Polyacetylenes, which are characteristic compounds of plants in this family, appear to be the most important group of bioactive compounds in goutweed [7]. Falcarinol and falcarindiol (Figure 5) are the main polyacetylene compounds present in extracts of this plant and also in small amounts in the essential oil [7,24,45,50,51,52,53]. They are found in all parts of goutweed, and their content increases in response to viral, fungal or bacterial infections [37,50]. These compounds are inhibitors of various enzymes, such as diacylglycerol acyltransferase (DGAT), inducible nitric oxide synthase (iNOS) and cholesteryl ester transfer protein (CETP) [54]. They exhibit biocidal, antifungal and antimicrobial activity, and may also have antiallergic [55] and anti-inflammatory effects [56].

### 3.4. Essential Oils

**Figure 6:**
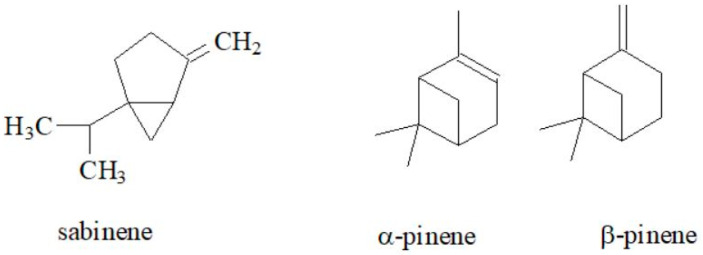

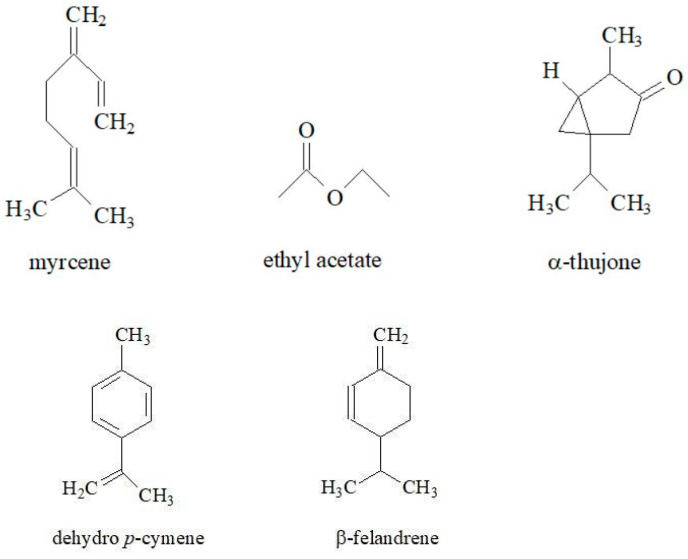
The chemical structures of sabinene, α-pinene, β-pinene, myrcene, ethyl acetate, α-thujone, dehydro-*p*-cymene and β-phellandrene.

Essential oils, due to their interesting and diverse properties, are widely used in many fields, including pharmaceuticals, antimicrobials, flavoring agents, cosmetics and fragrances [57,58,59]. The volatile components of essential oils are found in different morphological parts depending on the plant species [60]. In the Apiaceae family, the accumulation of essential oils occurs mainly in the secretory (oil) ducts, which are located along the vegetative and reproductive organs of plants [61]. The main components of the essential oils are mono- and sesquiterpenes [7]. Significant differences in the content of these compounds can be seen in the studies on goutweed essential oils. Their composition varies depending on the location of the plants, the stage of growth and the time of harvest [5,24,51]. Paramonov et al. [51] analyzed the composition of plant material and volatile compounds of goutweed, which is part of the Russian flora of the Iglinsky region. The main constituents were terpene compounds. Interestingly, sabinene was present in the highest proportion (about 63%), other components were α- and β-pinene (3.60% and 3.79%, respectively), myrcene (2.17%), ethyl acetate (4.82%), α-thujone (0.63%), dehydro-*p*-cymene (3.39%) and β-phellandrene (0.65%) (Figure 6).

**Figure 7:**
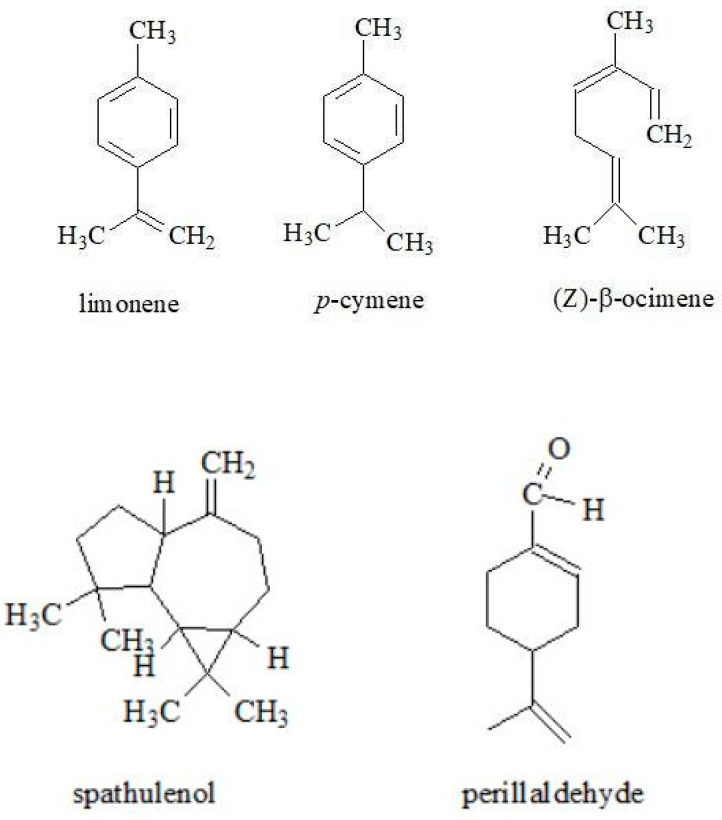

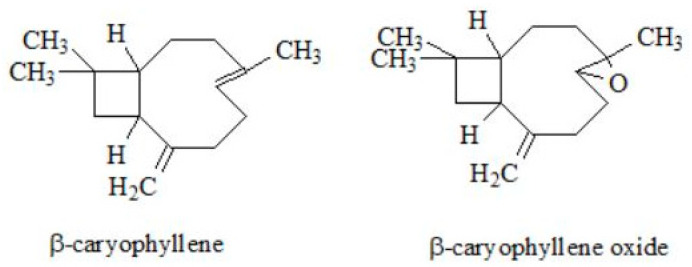
The chemical structures of limonene, *p*-cymene, (Z)-β-ocimene, spathulenol, perillaldehyde, β-caryophyllene and β-caryophyllene oxide.

In comparison, the essential oil of this species from the Central Balkans contained a wide variety of compounds, with the main components being α-pinene (13.3%), limonene (9.4%), *p*-cymene (8.8%), (Z)-β-ocimene (5.2%), β-pinene (5.0%), spathulenol (4.4%), perillaldehyde (4.1%), β-caryophyllene (3.9%) and β-caryophyllene oxide (3.4%) (Figure 7 presents the chemical structures of these compounds without α-pinene and β-pinene). In contrast, the content of sabinene—the main compound in goutweed essential oil from Russia—was relatively low at 1.8% [5]. Small amounts of falcarinol from the polyacetylene group were found in the essential oils of the leaves and stems of this plant: 0.6 and 0.2%, respectively [24].

## 4. Folk Medicine and Culinary Uses

**Figure 8:**
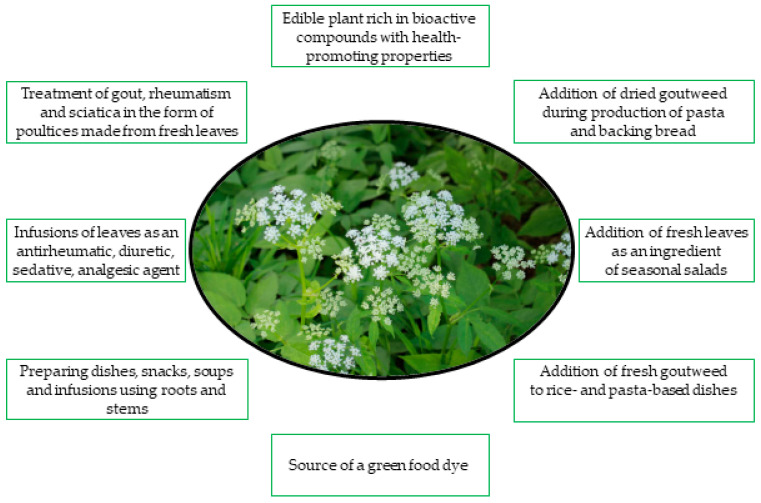
Traditional and contemporary medicinal and culinary uses of goutweed (*Aegopodium podagraria* L.).

Wild medicinal plants have been valued and used worldwide for their beneficial health effects and easy availability [1,62,63,64,65]. Their therapeutic potential has been known for thousands of years, and their medicinal uses have been passed down from generation to generation, playing an important role in the development of traditional treatments [66]. Goutweed has been used in folk medicine for centuries, and in the Middle Ages it was both cultivated as a medicinal plant and consumed as a vegetable. All parts of the plant show health-promoting effects (antirheumatic, diuretic, sedative and accelerating wound healing), although the leaves are most commonly used [10,25,67,68]. In the Middle Ages, goutweed was known as bishop’s weed, perhaps due to its use in the treatment of gout, often associated with an unhealthy diet, including excessive alcohol consumption, of the high clergy. At that time, it was also called St. Gerard’s herb [65,69,70,71,72,73]. St. Gerard of Toul (935–994) was the patron saint of gout sufferers, as reflected in the Latin name of the plant, podagraria (podagra = gout) [70,74]. As a remedy for gout, it was used by applying poultices of freshly crushed or bruised leaves to the affected joints [70]. The herb was also used in poultices and teas to relieve rheumatism and sciatica [74]. Herbalists still recommend goutweed as a diuretic and sedative, and drinking an infusion of the plant can help relieve joint pain and sciatica [10]. In Europe, wild edible plants have been and continue to be regarded as a dietary supplement due to their content of bioactive compounds and positive health effects [75]. Their importance increased in times when food was scarce, such as in times of war and famine. During World War II, wild plants were a valuable addition to the diet as a source of vitamins and minerals. One such plant was goutweed [23]. It was easy to obtain due to its adaptations to survive adverse environmental conditions [76,77] by developing a specialized morphological structure and synthesizing secondary metabolites such as polyphenols and vitamins [78]. Fresh young leaves were used in spring in salads and soups. Roots and stems were also used in the preparation of meals, snacks and infusions [7,23,75]. Today, the tradition of using wild plants is being revived [23,34,48,79,80,81]. The various traditional and contemporary uses of goutweed are shown in Figure 8.

## 5. Medicinal Properties

Plants of the Apiaceae family contain many bioactive compounds [24] and have a unique phytochemical composition that determines their health-promoting properties [31,82]. Goutweed is characterized by containing components with therapeutic effects [83]. Thanks to its rich chemical composition, it has the ability to exert a wide range of pharmacological effects [1,31]. Goutweed extracts have been shown to have antibacterial and antifungal [7,37,66,84], antioxidant [3,28,36,85,86], anti-inflammatory [7,45,50,87] and chemopreventive [87,88] properties. Scientific reports also describe antirheumatic, antimicrobial and hepatoprotective effects of goutweed extracts [1,3,38,53].

Alcoholic extracts of goutweed have been shown to be a natural source of antioxidants that help prevent oxidative damage. The radical scavenging activity was studied in extracts of *A. podagraria* L., obtained by heating under reflux at 50–55 °C for 3 h, filtering and concentrating using a rotary evaporator (stock solution concentration: 1 mg/mL). Ethanol extracts were shown to exhibit high antioxidant activity in both DPPH (IC_50_ = 66.135 ± 1.6 μg/mL) and ABTS (IC_50_ = 73.9 ± 8.7 μg/mL) radical scavenging assays. The radical scavenging activity of chloroform and ethyl acetate extracts obtained under the same conditions was significantly lower [3] Polyphenols have been found to be one of the main compounds responsible for the antioxidant properties [45]. Long-term consumption of foods rich in these compounds may have beneficial effects on conditions such as diabetes, cancer, osteoporosis, neurodegenerative diseases and cardiovascular disease. Polyphenols promote intestinal health and may act as potential prebiotics to support a range of probiotic functions, including reducing inflammation and preventing cancer by regulating intestinal flora [89]. Medicinal plants are also a source of anti-inflammatory compounds. Characteristic signs of inflammation include local redness, swelling, pain, warmth and loss of function such as stiffness and immobility [90]. Low-grade inflammation has been linked to several disorders and chronic diseases, including obesity, diabetes, cancer and cardiovascular disease [91,92,93,94,95,96,97,98]. Fresh goutweed juice has been used to treat various conditions, such as inflammatory diseases, gout, hemorrhoids and cancer [99]. Infusions of the leaves had anti-inflammatory and analgesic effects by suppressing inflammation [30,83]. The anti-inflammatory activity of goutweed can be attributed to the polyacetylenes falcarinol and falcarindiol [52]. These compounds are potent inhibitors of lipoxygenases (5-, 12- and 15-lipoxygenases), which are involved in the processes of cancer progression and atherosclerosis. Falcarindiol effectively inhibits cyclooxygenases, particularly 1-cyclooxygenase, whereas the cyclooxygenase inhibitory activity of falcarinol is not as pronounced [100,101]. In an in vitro study, falcarindiol isolated from goutweed root was found to have an IC_50_ = 0.3 μM in the COX-1 assay, while indomethacin has an IC_50_ = 9 μM. Comparison of these values indicates that falcarindiol is 30 times more active than indomethacin, a well-known anti-inflammatory and antiarthritic drug [52]. Anti-inflammatory activity has also been attributed to flavonoids. Flavonoids such as quercetin, genistein, apigenin, kaempferol and epigallocatechin-3-gallate have been shown to inhibit the secretion of enzymes such as lysozymes and β-glucuronidases, leading to a reduction in inflammatory responses. In addition, flavonoids modulated the expression and activation of cytokines such as IL-1β, TNF-α, IL-6 and IL-8; they also regulated the gene expression of many pro-inflammatory molecules [102].

Inflammation is often accompanied by pain, which can be successfully controlled using traditional medicinal plants. Pain is usually initiated by a noxious stimulus and transmitted to the central nervous system through neural networks. It is a protective mechanism that helps protect the body from a potential injury and indicates the need to respond to potential threats [103]. Medicinal plants have been used as painkillers since ancient times. Their action contributes to the restoration of the biological balance, thanks to their natural active ingredients, synergistic action and low accumulation in the body [104]. In some cases, plants and their products could be ideal substitutes for synthetic drugs [105]. Flavonoids are known to have anti-inflammatory effects, reducing joint pain and inflammation [102]. A study of the properties of goutweed showed some effect on anxiety and the ability to suppress cyclooxygenase-1, which may be one of the possible mechanisms of its analgesic effect [106].

For centuries, various plants have been used to treat many bacterial infections [107] and there are many reports on the antibacterial properties of plant extracts [3,66]. Goutweed extracts are a source of bioactive compounds, including secondary metabolites with antimicrobial activity [66]. Among them are flavonoids, which show potential antimicrobial activity, synergism with antibiotics and suppression of bacterial virulence [108], as well as antiviral properties [109]. Quercetin has been found to have antiviral activity at various stages of the infection. Several studies have highlighted the potential use of quercetin as an antiviral agent due to its ability to suppress the early stages of the viral infection, interact with viral replication proteases and reduce inflammation caused by infection [110]. Orhan et al. [111] described the antibacterial and antifungal effects of rutin against eight strains of bacteria and their drug-resistant isolates, and fungi. They observed strong antimicrobial activity of rutin and other flavonoids. The activity of rutin against *E. coli*, *P. auruginosa*, *S. aureus*, *K. oxytoca* and *B. subtilis*, and its antifungal activity against the fungus *C. albicans*, have been confirmed [112]. All of these compounds are present in goutweed.

Given the growing resistance of bacteria to antibiotics and the possibility of the plasmid-mediated transfer of resistance genes, it is necessary to search for new sources of antimicrobial agents. For this reason, scientists are investigating biologically active compounds from plants as potential antimicrobial agents [66]. The ethanol extract of goutweed has been shown to have antimicrobial activity and to produce synergistic and additive effects with antibiotics. The antibacterial activities of aqueous, ethanolic and ethyl acetate extracts of goutweed were tested in vitro against six human-pathogenic bacteria: *Bacillus mycoides*, *Bacillus subtilis*, *Staphylococcus aureus*, *Enterobacter cloacae*, *Klebsiella pneumonia* and *Pseudomonas fluorescens*. The ethanolic extract showed the highest activity (MIC: 1.25–5 mg/mL) and was chosen to investigate the effects of its combinations with antibiotics (streptomycin and chloramphenicol) using the checkerboard method. Synergistic and/or additive interactions of this extract with both antibiotics were found against the bacteria tested. Synergism was observed against *B. subtilis*. These results may contribute to the use of antibiotics in lower doses [25]. Aqueous, ethanolic and ethyl acetate extracts of goutweed also inhibited the growth of some phytopathogenic bacteria (*Agrobacterium radiobacter* pv. *tumefaciens*, *Erwinia carotovora*, *Pseudomonas fluorescens* and *Pseudomonas glycinea*). The antimicrobial properties of aqueous, ethanolic and ethyl acetate extracts were evaluated using the disk diffusion method at a concentration of 15 mg of dry extract per disk. The results indicate that they could potentially be used as the biological pesticides [113]. Furthermore, ethanolic extracts of this plant showed inhibitory effects against the growth of *Staphylococcus aureus*. Extracts obtained from different parts of goutweed (leaves, flowers, seeds and rhizomes) were active against both a reference (ATCC 29213) and a clinical strain of *S. aureus* (MIC = 64 mg/mL). Therefore, they can be considered as a potential additive to formulations used to treat infections caused by these bacteria [114]. Herbal products are also being studied as adjunctive therapies for various mental disorders. Scientists are focusing on confirming the psychoactive properties of medicinal plants; as a result, compounds that affect the central nervous system have been identified. Some of these are now being used as ingredients in pharmaceutical products [115]. Bioactive compounds such as saponins [116], alkaloids [117,118], polyphenols [119], triterpenoids [120], essential oils [121,122], fatty acids [123] and flavonoids [124] have anxiolytic and antidepressant properties. Many of the active ingredients in goutweed have been shown to affect the central nervous system [106]. Ukrainian researchers used male and female mice to investigate the effects of extracts from the aerial parts of goutweed on levels of depression and anxiety, locomotor activity, exploratory behavior and memory. It was shown that the extract at a dose of 100 mg/kg reduced depression levels in the tail suspension test in females and reduced anxiety in the cross maze test in both sexes. The efficacy of the extract in mice was dose and sex dependent [106]. According to Takeda et al. [125] and Tsuji et al. [126], caffeic acid exhibits antidepressant and/or anxiolytic effects. Falcarindiol and ferulic acid have an affinity towards serotonin receptors [127], whose disruption may be involved in the etiology of anxiety, depression and schizophrenia. Additionally, animal studies have shown that adequate intake of minerals such as potassium and magnesium is associated with reduced levels of depression and mood disorders [128]. A sedative effect of goutweed is mentioned in traditional medicine but has not yet been scientifically proven [106]. At the same time, goutweed preparations have been proven to support the treatment of depression [39,129].

The complex composition of unprocessed herbal material is an important factor in biological activity, including metabolic effects. However, there is a risk of toxic effects or undesirable interactions with concomitant medicines or foods [38]. Polyacetylenes found in common goutweed are secondary metabolites that have been found to be undesirable in food due to their toxicity. Some can cause severe skin sensitization and are neurotoxic in high concentrations. They have strong selective cytotoxic activity against cancer cells. Almost all polyacetylenes found in the Apiaceae family are of the falcarinol type. Falcarinol can cause some neurotoxic effects, but no toxicity of this compound has been reported to date. Side effects are only seen at very high doses, which can also cause allergic reactions [50]. The low toxicity of goutweed preparations is a desirable feature, especially when used as a food and fodder crop [7]. Scientific studies confirm the hypolipidemic properties of both aqueous extracts and tinctures obtained from the aerial parts of this plant and their sufficient level of safety [38]. The mechanism of action is complex and is due to the diverse composition of substances of herbal origin, such as herbal extracts and tinctures [39]. Goutweed, consumed as a food, can exert significant cytotoxic effects on human prostate cancer cells. An MTT cell viability assay was conducted to investigate the cytotoxic effects of *A. podagraria* extracts on human prostate cancer (PC3), colorectal cancer (HCT116) and lung cancer (A549) cells. For all cell types, the observed cytotoxic effects increased in a dose-dependent manner and the highest activity was found in the case of PC3 cells. The research suggests that this plant and its bioactive compounds may help treat several types of cancer, especially prostate cancer [130].

There is a growing interest in traditional medicine in finding new hepatoprotective agents that can help mitigate or reverse liver damage [124]. A study of mice given goutweed leaf extracts showed that the activity of cytolysis markers was lower than in control animals; this could indicate a beneficial effect on liver health. The mechanism of action may be related to the ability to bind free radicals and stabilize cell membranes, thanks to the phenolic compounds present in the extract. The extract of goutweed herb had a beneficial effect on the course of acute toxic hepatitis, thus showing a hepatoprotective effect [131]. The hepatoprotective effect of phytopreparations was also confirmed by a study carried out on mice on the effect of biologically active substances found in the roots, leaves and flowers of goutweed. Extracts from these parts showed a protective effect on the liver of mice [82]. Both the infusion, extract and trifolin were effective against cytolysis, as confirmed by a decrease in plasma enzyme activity and the ratio of aspartate aminotransferase to alanine aminotransferase [132]. The results suggest that goutweed extracts can be used to support the treatment and prevention of diseases associated with liver damage. These extracts can also expand the pool of hepatoprotectors and individualize pharmacotherapy [131].

Plant-derived nephroprotective compounds can alleviate interstitial nephritis, intraglomerular hemodynamics, renal tubular necrosis and glomerulonephritis [133]. It is important to remember that plants with nephroprotective potential should be considered as an adjunct to treatment, not as a replacement treatment [134,135]. Plants with nephroprotective effects can reduce drug toxicity when taken together and can help protect the kidneys from the harmful effects of certain drugs. Incorporating plants with nephroprotective effects into pharmacotherapy can increase the efficacy of treatment while minimizing potential renal toxicity [136]. The rich chemical composition of goutweed explains the wide range of its pharmacological activity, including its beneficial effects on the kidneys [132]. The plant has mainly been used to treat urinary and renal diseases [36]. Extracts from the aerial parts showed protective effects on the kidneys and liver. In particular, they were effective in preventing kidney damage caused by ischemia, myoglobinemia, gentamicin, ethylene glycol and carbon tetrachloride. The extracts were helpful in reducing mortality and histological changes in the kidneys, restoring urine thickening functions and reducing proteinuria and azotemia [38]. Goutweed leaf extract contains active components such as a protein–polysaccharide complex and the flavonoid trifolin, which are known for their nephroprotective effects. These compounds were found to be helpful in reducing mortality, azotemia and proteinuria; eliminating amenorrhea; preventing the decrease in glomerular filtration rate; and normalizing the histopathological structure of the kidneys [137]. Goutweed extracts also supported renal excretory function in rats with impaired renal function without causing hyperkalemia, despite their high potassium content [138]. The aqueous extract and tincture of the aerial part of goutweed have been shown to improve renal excretory function and have a positive effect on uric acid exchange. The dry extract and tincture of the aerial part of goutweed showed significant nephroprotective and hepatoprotective activity, as well as beneficial effects on purine and carbohydrate metabolism [39].

Goutweed has been of interest to Polish researchers for its potential use in cosmetology. Extracts were made from the aerial parts with a mixture of water and glycerol (80:20) and tested for antioxidant potential, as well as the activity of collagenase and elastase—enzymes responsible for skin aging. Studies in human skin cell lines showed that a 5% extract of goutweed inhibited both enzymes by 70%. In addition, the extract had a beneficial effect on cell proliferation of keratocytes and fibroblasts. These abilities indicate the potential of goutweed as a cosmetological plant material with antiaging and skin-protective properties [36].

The wide spectrum of biological activity of goutweed and its low toxicity make it a valuable plant with a range of possible beneficial effects on health. Table 2 shows studies on the bioactivity of *Aegopodium podagraria* L. in different research models.

## 6. Conclusions

Goutweed is a common plant in the temperate climate zone. All morphological parts of the plant are edible, with the leaves being the most commonly used. In Poland, dried goutweed leaves are commercially available and used, for example, as an infusion (herbal tea). The health-promoting properties of this plant have been known and used in folk medicine for centuries. Since then, they have been confirmed by scientific research, mainly conducted in Europe, both in vitro and in vivo and also on cell lines. Today, we know that extracts of the plant have very interesting properties such as anti-inflammatory, antimicrobial, anticancer, antioxidant, metabolic and antiaging. It would be expedient and valuable to confirm these properties in clinical studies, which unfortunately have not yet been conducted. In recent years, there has been a trend towards the development of functional foods. Such foods not only provide nutrients but also enrich the daily diet with health-promoting components. One plant that has found its place in this area is goutweed. Its dried aerial parts have been used in the production of bread and pasta, enriching these products with health-promoting bioactive compounds such as antioxidants, minerals and vitamins. Given its easy accessibility and valuable chemical composition, goutweed can be a valuable source of health-promoting compounds and is worth adding to the daily diet.

## Figures and Tables

**Table 1:** The nutritional composition of the aerial parts of goutweed.

Component	Part of the Plant	Content	References
Macroelements:			
K	Leaves	38,372 (μg/g)	[24]
	Stems	76,848 (μg/g)	
	Aerial parts	42,263 (μg/g)	[34]
Mg	Leaves	2233 (μg/g)	[24]
	Stems	2082 (μg/g)	
	Aerial parts	1598 (μg/g)	[34]
Ca	Aerial parts	6632 (μg/g)	[34]
P	Aerial parts	2678 (μg/g)	[34]
Microelements:			
Fe	Aerial parts	38.20 (μg/g)	[34]
Zn	Leaves	39 (μg/g)	[24]
	Stems	24 (μg/g)	
Cu	Leaves	3.7 (μg/g)	[24]
	Stems	1.85 (μg/g)	
	Aerial parts	14.16 (μg/g)	[34]
Mn	Leaves	32 (μg/g)	[24]
	Stems	20.5 (μg/g)	
	Aerial parts	32 (μg/g)	[34]
Se	Aerial parts	0.21 (μg/g)	[34]
Cr	Leaves	514 (ng/g)	[24]
	Stems	135 (ng/g)	
	Aerial parts	0.93 (μg/g)	[34]
Co	Leaves	28 (ng/g)	[24]
	Stems	4.8 (ng/g)	
Pb	Leaves	484 (ng/g)	[24]
	Stems	341 (ng/g)	
Vitamins:			
Ascorbic acid (vit. C)	Aerial parts	46.5 (mg/100 g)	[34]
Carotene (provit. A)	Aerial parts	0.21 (mg/100 g)	[34]
Thiamin (vit. B1)	Aerial parts	0.016 (mg/100 g)	[34]
Riboflavin (vit. B2)	Aerial parts	0.11 (mg/100 g)	[34]
Nutritional components:			
Protein	Aerial parts	51.30 (mg/g)	[35]
Proline	Aerial parts	21.07 (µmol/g)	[35]
Total free amino acids	Aerial parts	29.62 (µg/g)	[35]
Glucose	Aerial parts	55.92 (mg/100 g)	[35]
Sucrose	Aerial parts	14.99 (mg/100 g)	[35]
Total soluble carbohydrate	Aerial parts	242.7 (mg/100 g)	[35]
Other components:			
Chlorophyll	Aerial parts	20.51 (mg/100 g)	[35]
Total carotenoids	Aerial parts	20.88 (mg/100 g)	[35]
β-Carotene	Aerial parts	165.91 (µg/100 g)	[35]
		11.12 (mg/100 g)	[29]
Lycopene	Aerial parts	236.63 (µg/100 g)	[35]
Flavonoids	Aerial parts	12.71 (mg/100 g)	[35]
Anthocyanins	Aerial parts	16.01 (mg/100 g)	[35]
P-active substances (the sum of catechins and flavonoids in terms of rutin)	Aerial parts	17.3 (mg/100 g)	[34]

**Table 2:** Studies on the bioactivity of *Aegopodium podagraria* L. in different research models.

Part of Plant (Origin)	Plant Material Formulation	Research Model/Method	Aim of Study	Results	Ref.
Antimicrobial activity					
Aerial parts (Serbia)	Extracts (water, ethanol, ethyl acetate)	Bioassay—human-pathogenic bacteria (disk diffusion and tube dilution methods)	Evaluation of antibacterial activity against: *B. mycoides*, *B. subtilis*, *S. aureus*, *E. cloacae*, *K. pneumonia*, *P. fluorescens*	Ethanolic extract showed highest antibacterial activity and synergistic and/or additive effects with antibiotics Synergism was observed against *B. subtilis*	[25]
Aerial parts Rhizomes (Poland)	Extracts (hexane, ethyl acetate, water)	Bioassay—human-pathogenic bacteria (serial dilution method in fluid medium)	Evaluation of antibacterial and antifungal activity against *S. aureus*, *E. faecalis*, *E. coli*, *K. pneumoniae*, *P. aeruginosa*, *C. albicans*, *M. gypseum*	Different antimicrobial activity, depending on part of the plant and extractant	[139]
Leaves Flowers Seeds Rhizomes (Poland)	Extract (ethanol)	Bioassay—*S. aureus* culture on petri dishes (disk diffusion method)	Evaluation of antistaphylococcal activity against reference and clinical strain of *S. aureus*	Inhibiting activity against tested strains of *S. aureus*	[114]
Rhizomes (England probably)	Extract (acetone) separated into two fractions containing falcarinol and falcarindiol	Bioassay—spore germination tests, using Butt slides and impregnated agar plugs	Evaluation of antifungal activity against 10 fungal strains, including *A. brassicicola*, *B. cinerea*, *S. nodorum* and others	Falcarindiol is a major antifungal constituent, responsible for inhibiting the growth of tested fungi	[101]
Seeds (Poland)	Extract (ethyl acetate)	Bioassay—fungal culture on petri dishes and experiments in field conditions	Evaluation of suitability for plant protection against phytopathogenic fungi: *F. culmorum*, *B. cinerea, M. penicullata*	*A. podagraria* seed extract had a low effect on the growth of tested fungi	[140]
Antioxidant activity					
Aerial parts (Bulgaria)	Extracts (chloroform, ethanol, ethyl acetate)	DPPH and ABTS radical scavenging activity—in vitro assays	Determination of radical scavenging activity of extracts obtained using different solvents	Ethanolic extract exhibited highest antioxidant potential in both assays	[3]
Aerial parts (Poland)	Extracts (ethanol-water 8:2, obtained in various conditions)	DPPH radical scavenging activity assay performed by reversed-phase high-performance liquid chromatography (DPPH-RP-HPLC)	Evaluation of influence of plant preparation method and extraction conditions on antioxidant potential of extracts	Extract prepared from dry plant using ultrasonic bath showed highest antioxidant potential	[85]
Leaves Rhizomes Seeds Flowers (Poland)	Extracts (ethanol or acetone, obtained by various techniques)	DPPH radical scavenging activity—in vitro assay	Investigation of DPPH scavenging activity of extracts from various parts of the plant obtained using different extracting methods and solvents	All parts of the plant exhibit radical scavenging activity, depending mainly on the extraction technique and extraction time	[53]
Leaves (Poland)	Extracts (ethanol, water)	DPPH radical scavenging activity, FRAP (Ferric Reducing Antioxidant Power), TPC (Total Phenolic Content)—in vitro assays; Cell Cultures THP-1	Evaluation of antioxidant potential (DPPH, FRAP), total polyphenol content (TPC) and effects against fluoride-modulated oxidative stress in THP-1 cell line	Extracts have antioxidant activity, promote antioxidant enzymes and provide a protective effect against sodium fluoride toxicity	[45]
Anti-inflammatory activity					
Roots Leaves Stems Flowers (Denmark)	Extracts (water, methanol, acetone, dichloromethane, ethyl acetate hexane)	COX-1 in vitro assay	Screening in vitro for cyclooxygenase-1 (COX-1) inhibitory activity	The highest activity was observed for hexane extract of flowers. Other extracts, except aqueous, also showed activity. The high level of COX-1 inhibitory activity is related to falcarindiol content. Results indicate the potential use of goutweed in herbal medicine	[52]
Anticancer activity					
Aerial parts (Turkey)	Extracts (water, methanol)	MTT cell viability assay—human cell cultures: - prostate cancer - colorectal cancer - lung cancer	Evaluation of cytotoxicity	Considerable cytotoxic effects on human prostate cancer cells. May help treat prostate cancer	[130]
Impact on physical endurance and nervous system					
Aerial parts (Ukraine)	Extracts (water, ethanol)	Male and female mice	Evaluation of the effects of extracts on levels of depression and anxiety, locomotor activity, exploratory behavior and memory	Beneficial effects of extracts on the CNS in mice, including antidepressant effect and reduction in signs of anxiety	[106]
Aerial parts (Ukraine)	Dry extract, tincture	Mice and rats: weight-loaded forced swimming test, extrapolation escape test, reserpine-induced depression model	Verification of pharmacological effects of goutweed on physical and mental condition of animals: mice and rats	Heterogeneous results depending on extract type and animal model	[141]
Metabolic effects					
Aerial parts (Ukraine)	Extracts (water, ethanol)	Male rats	Evaluation of effects on electrolyte, glucose and uric acid metabolism	Ethanolic extracts exert hypoglycemic effects in a metabolic syndrome-like model Further study of extract dosage regimens required	[142]
Aerial parts (Ukraine)	Extracts (water, ethanol)	Male rats	Evaluation of effects of extracts on carbohydrate and protein metabolism, as well as urea and plasma enzyme activity, in rats treated with a single dose of ethanol	Extracts did not induce unfavorable shifts in total protein, albumin, uric acid and creatinine content (only a moderate increase in urea level was observed). Results confirm the safety of goutweed preparations	[143]
Aerial parts (Ukraine)	Extract (ethanol)	Outbred male albino rats	Evaluation of metabolic effects of extracts and their combinations with metformin in dexamethasone-treated rats	Beneficial effects of the combination of ethanolic extract and metformin: reduction in plasma ALT activity, increase in urea clearance and normalization of ALP activity	[2]
Aerial parts (Ukraine)	Ethanol extract	Outbred male albino rats	Assessing the effect of goutweed tincture combined with metformin on renal excretory function and state of mineral metabolism in dexamethasone-treated rats	Metformin therapy combined with ethanolic extract resulted in reduction of proteinuria and enzymuria, and normalization of potassium level in blood	[144]
Aerial parts (Ukraine)	Extracts (water, ethanol)	Wistar male albino rats	Evaluation of the influence of extracts and their combinations with metformin on renal excretory function in rats fed an atherogenic diet combined with protamine sulfate	Goutweed extract and tincture normalize lipid composition of liver in rats with lipid and carbohydrate metabolism disorders caused by protamine sulfate and an atherogenic diet. Tincture also exerts a permissive effect on metformin action on glucose metabolism, but not on lipid metabolism	[145]
Aerial parts (Ukraine)	Extracts (water, ethanol)	Female rats	Determination of the influence of extracts on renal function and metabolic processes in rats receiving hydrochlorothiazide	Results substantiate the potential use of goutweed extracts in combination with hydrochlorothiazide. In addition, the extract increased uric acid excretion and decreased plasma urea levels	[146]
Flowers (Ukraine)	Essential oil	Male albino mice	Diuretic and uricosuric activity of goutweed flower essential oil	Results include increased excretion of creatinine, urea and uric acid with unchanged urine volume, suggesting that essential oil of flowers may be involved in the diuretic and uricosuric activity of goutweed	[132]
Antiaging activity					
Aerial parts (Poland)	Extract (water/glycerol, 80:20)	Cell culture: HaCaT (normal human keratinocytes)	Determination of anticollagenase activity and antielastase activity by scratch wound assay	Beneficial effects on skin cells: inhibition of elastase and collagenase, stimulation of keratinocyte and fibroblast migration, with a potentially significant impact on delaying skin aging	[36]

## Data Availability

No new data were created or analyzed in this study. Data sharing is not applicable to this article.
